# miR-380-5p-mediated repression of TEP1 and TSPYL5 interferes with telomerase activity and favours the emergence of an “ALT-like” phenotype in diffuse malignant peritoneal mesothelioma cells

**DOI:** 10.1186/s13045-017-0510-3

**Published:** 2017-07-17

**Authors:** Graziella Cimino-Reale, Paolo Gandellini, Francesca Santambrogio, Marta Recagni, Nadia Zaffaroni, Marco Folini

**Affiliations:** Molecular Pharmacology Unit, Department of Applied Research and Technological Development, Fondazione IRCCS Istituto Nazionale dei Tumori, Via Amadeo, 42 - 20133 Milano, Italy

**Keywords:** Alternative lengthening of telomeres, Diffuse malignant peritoneal mesothelioma, miRNA, Telomerase associated protein 1, Telomere maintenance mechanisms, Testis-specific protein, Y-encoded-like 5

## Abstract

**Background:**

Understanding the molecular/cellular underpinnings of diffuse malignant peritoneal mesothelioma (DMPM), a fatal malignancy with limited therapeutic options, is of utmost importance for the fruitful management of the disease. In this context, we previously found that telomerase activity (TA), which accounts for the limitless proliferative potential of cancer cells, is prognostic for disease relapse and cancer-related death in DMPM patients. Consequently, the identification of factors involved in telomerase activation/regulation may pave the way towards the development of novel therapeutic interventions for the disease. Here, the capability of miR-380-5p, a microRNA negligibly expressed in telomerase-positive DMPM clinical specimens, to interfere with telomerase-mediated telomere maintenance and, hence, with cancer cell growth was assessed on preclinical models of DMPM.

**Methods:**

DMPM cells were transfected with a miR-380-5p synthetic precursor, and the effects of miRNA replacement were evaluated in terms of growing capability, induction of apoptosis and interference with TA. Reiterated weekly transfections were also performed in order to analyse the phenotype arising upon prolonged miR-380-5p reconstitution in DMPM cells.

**Results:**

The ectopic expression of miR-380-5p elicited a remarkable inhibition of TA and resulted in DMPM cell growth impairment and apoptosis induction. In particular, we demonstrated for the first time that these effects were the result of a molecular circuitry converging on telomerase associated protein 1 (TEP1), where the miRNA was able to target the gene both directly in unconventional targeting modality and indirectly via p53 accumulation consequent to miRNA-mediated downregulation of testis-specific protein, Y-encoded-like 5 gene. Moreover, miR-380-5p did not cause telomere attrition and cell growth arrest in long-term DMPM transfectants, which in turn showed slightly elongated telomeres and molecular features (e.g. c-circle DNA and reduced expression levels of chromatin remodeler ATRX) resembling an alternative lengthening of telomeres (ALT) phenotype.

**Conclusions:**

miR-380-5p interferes with TA in DMPM cells by targeting TEP1. Notably, in the long-term setting, miR-380-5p-mediated impairment of TA did not result in telomere attrition. Instead, a phenotype reminiscent of ALT emerged in DMPM cells as possible compensatory pathway that safeguards DMPM cell growth, an event that may be regarded as a potential resistance mechanism to anticancer therapies based on telomerase inhibitors.

**Electronic supplementary material:**

The online version of this article (doi:10.1186/s13045-017-0510-3) contains supplementary material, which is available to authorized users.

## Background

Telomeres are guanine-rich DNA (5′-TTAGGG-3′) repeats and associated proteins located at the very end of eukaryotic chromosomes [1]. Telomere maintenance mechanisms (TMM) endow tumour cells with replicative immortality—a hallmark of cancer—in that they counteract the progressive telomere attrition, which occurs during each cell replication cycle and limits normal cell divisions to a finite number [1].

**Fig. 1:**
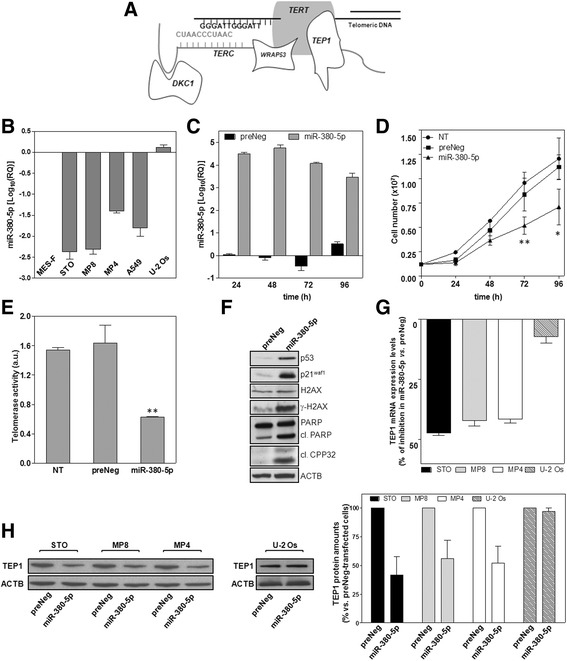
miR-380-5p impairs the growth of DMPM cells and inhibits TA by downregulating TEP1. **a** The drawing schematically shows the minimal components TERT (telomerase reverse transcriptase) and TERC (telomerase RNA component) of telomerase associated with telomeric DNA and with some ancillary proteins involved in the stabilization (DKC1: dyskerin pseudouridine synthase 1), proper functioning (TEP1, telomerase associated protein 1) and intra-nuclear localization (WRAP53, WD repeat containing antisense to TP53) of the enzyme. **b** Basal miR-380-5p expression levels in normal, non-immortalized mesothelial (MES-F) cells, in TA-positive DMPM (STO, MP8, MP4) and lung adenocarcinma (A549) cells as well as in the TA-negative (ALT-positive) U-2 Os cell line (Log_10_(RQ) with respect to MES-F cells; mean values ± s.d.). **c** Time-course assessment of miR-380-5p expression levels in preNeg- and miR-380-5p-transfected STO cells (Log_10_(RQ) with respect to non transfected (NT) cells; mean value ± s.d.). **d** Growth curves of NT, preNeg- and miR-380-5p-transfected STO cells (number of growing cells; mean value ± s.d.); *P < 0.05; **P < 0.02 in miR-380 vs. preNeg. **e** Quantification of TA (arbitrary units (a.u.); mean values ± s.d.); **P < 0.01 vs. preNeg-transfected cells. **f** Representative immunoblotting showing the amounts of p53, p21^waf1^ and of apoptotic markers upon a 96-h transfection of STO cells with preNeg or miR-380-5p. Cropped images of selected proteins are shown. **g** TEP1 expression levels in miR-380-5p- vs*.* preNeg-transfected DMPM and U-2 Os cells (percentage of gene expression inhibition; mean values ± s.d.). **h** Representative immunoblotting (cropped images of selected proteins are shown) and quantification of TEP1 amounts in preNeg- and miR-380-5p-transfected DMPM and U-2 Os cells. Data have been reported as percentage of protein amounts in miR-380-5p- *vs.* preNeg-transfected DMPM and U-2 Os cells (mean values ± s.d.)

Telomerase activity (TA) and the alternative lengthening of telomere (ALT) pathway are the only two TMM yet identified in human cancers [1]. Telomerase is a reverse transcriptase consisting of two main subunits: the long non-coding RNA molecule TERC, which contains the template sequence for the synthesis of telomeric DNA, and the catalytic protein portion telomerase reverse transcriptase (TERT) [1]. The two subunits associate with distinct ancillary proteins that are necessary for the correct assembly, stability, mode of operation and intracellular trafficking of the holoenzyme (Fig. 1a) [1].

TA is the most frequently activated TMM in human tumours, whereas ALT is prevalently activated in mesenchymal tumours (e.g. osteosarcomas, soft-tissue sarcomas) and, less frequently, in carcinomas (breast, gastric, ovarian cancers), malignant melanomas and neuroendocrine pancreatic cancers [2].

Although ALT is thought to be driven by homologous recombination, the knowledge of the molecular mechanisms underpinning it in cancers is still fragmentary. Consequently, ALT tumours are generally defined according to a combination of two or more markers [2], including *i)* the presence of long and heterogeneous telomeres and of extrachromosomal telomeric DNA (e.g. c-circle DNA); *ii)* the occurrence of spontaneous and telomeric-localized DNA damage; *iii)* the presence of ALT-associated promyelocytic leukaemia (PML) bodies (APB), a subset of PML nuclear bodies containing telomeric DNA and telomere-associated proteins/DNA repair factors; *iv)* the absence of TA and *v)* the loss/reduced expression and/or mutations of the chromatin remodeler ATRX [3, 4].

Translational studies have revealed that TMM can variably affect the outcome of patients as a function of tumour type. For instance, it has been demonstrated that patients with ALT-positive, high-grade glioblastoma multiforme have significantly longer survival than those with ALT-negative tumours, whereas a poor prognosis characterizes patients with ALT- and TA-positive osteosarcomas with respect to patients with tumours that apparently lack of TMM [1]. In addition, ALT and TA have been associated with an unfavourable disease outcome in liposarcomas, although a worse prognosis, in terms of increased mortality, was observed for patients with ALT tumours [1]. Moreover, TA was found to be prognostic for tumour relapse and cancer-related death in diffuse malignant peritoneal mesothelioma (DMPM) as well as for cancer-related death in malignant peripheral nerve sheath tumour patients, whereas ALT failed to significantly affect clinical outcome in both cancer types [5, 6].

The fragmentary information currently available on ALT at molecular level has thus far hampered the development of ALT-specific therapeutic interventions, whereas distinct anti-telomerase therapeutic approaches have been developed and presently tested in clinical trials [7]. This evidence suggests that improving the knowledge of the molecular mechanisms subtending the activation/regulation of ALT vs*.* telomerase and vice versa during cancer development and progression is of utmost importance to better understand the implications of telomere biology and of telomerase-based therapeutic approaches in clinical tumours.

MicroRNAs (miRNAs) are small non-coding RNAs that negatively regulate gene expression through the RNA interference (RNAi) pathway [8]. They are involved in the control of several biological processes, and their deregulated expression may contribute to diverse pathological conditions, including cancer development and progression [8]. Accumulating evidence suggests that, compared to normal counterparts, tumour tissues show typical miRNA signatures, which may represent useful diagnostic markers. Moreover, changes in the expression levels of specific miRNAs have been documented to be predictive of prognosis or treatment response in cancer patients [9].

Deregulated miRNA levels trigger a global perturbation in the expression or function of target genes having pro- or anti-tumour properties. This implies that a specific miRNA may exert oncogenic or tumour-suppressive functions depending on the cell/tissue context and the presence of its target genes [1]. Consequently, attempts to modify the expression of selected miRNAs currently represent an intriguing approach to defeat cancer as well as to ameliorate the response of tumour cells to standard anticancer therapies [8, 9].

Owing to their role in cancer, miRNAs may plausibly contribute to the establishment and/or regulation of TMM. Here, we show that miR-380-5p, a miRNA that is negligibly expressed in telomerase-positive DMPM tissues and cell lines, interferes with telomerase-dependent telomere maintenance. In particular, the ectopic miR-380-5p expression in DMPM cells results in TA inhibition through a molecular circuitry involving the downregulation of TEP1 and TSPYL5, which we demonstrate for the first time to be direct targets of the miRNA. In addition, we found that miR-380-5p may lead to the emergence of molecular features reminiscent of an ALT phenotpye, as long-term DMPM cell cultures expressing the miRNA were characterized by reduced expression levels of ATRX and increased levels of telomeric c-circle DNA.

## Methods

### Reagents and chemicals

miR-380-5p synthetic precursor (pre-miR-380-5p) and the negative control oligomer (preNeg) were purchased as Pre-miR™ miRNA precursor molecules (Thermo Fisher Scientific). miR-380-5p inhibitor (LNA380) and the relative control oligomer (LNANeg) were purchased as miRCURY LNA™ microRNA Inhibitors (Exiqon A/S, Vedbaek, Denmark). Commercially available small interfering RNAs (siRNA) targeting TEP1 (siTEP1), TSPYL5 (siTSPYL5), p53 (sip53) and control siRNAs (siCTR) were obtained from Santa Cruz Biotechnology Inc. (Dallas, TX, USA). Target protector oligomers were purchased as miScript Target Protectors (Qiagen, Hilden, Germany). Doxorubicin hydrochloride was from Sigma-Aldrich (Milano, Italy). The reagents were dissolved in nuclease-free, sterile water, stored at −20 °C and diluted at the appropriate working concentration just before use.

### Cell lines and transfection procedures

DMPM (STO, MP8 and MP4) cells [10], A549 lung cancer cells (ATCC®-CCL-185™), U-2 Os osteosarcoma cells (ATCC® HTB-96™) and human adult mesothelial MES-F cells (Zen-Bio Inc., Research Triangle Park, NC) were maintained in the logarithmic growth phase in Dulbecco’s modified Eagle’s (DMEM), DMEM-F12 (Lonza Milano S.r.l, Treviglio, Italy) and MSO-1 (Zen-Bio Inc.) media, respectively, supplemented with foetal bovine serum, at 37 °C in a humidified incubator at 5% CO_2_. Cells were periodically monitored for DNA profile by short tandem repeats analysis using the AmpFISTR Identifiler PCR amplification kit (Thermo Fisher Scientific, Monza, Italy).

For the transfection procedures, cells seeded at the appropriate density and allowed to attach for 72 h (DMPM cells) or 24 h (A549, U-2 Os, MES-F) were incubated with Lipofectamine-2000^TM^ (Thermo Fisher) in Opti-MEM I (Thermo Fisher). After 15 min at r.t., lipid-treated cells were added with Opti-MEM I medium containing the oligomers at the final concentrations of 20 nM (pre-miR-380-5p; preNeg), 100 nM (LNA380; LNA-Neg) or 50 nM (siTEP1, siTSPYL5 and sip53), and incubated for 4 h at 37 °C. For target protection experiments, 100 nM of custom designed TEP1 or TSPYL5 target protectors was transfected in combination with miR-380-5p precursor or preNeg, under the same transfection conditions described above. Cells were then harvested according to the timeline of each experiment and subsequently analysed. For the long-term assessment of miR-380-5p effects, the transfection procedure was reiterated every 7 days, up to 3 months. For the assessment of cell growth, cells were collected at different time intervals and their number and viability were evaluated by a dye exclusion test (0.4% Trypan Blue Solution, Sigma-Aldrich) using a TC20^TM^ automated cell counter (Bio-Rad Laboratories S.r.l., Segrate, Italy). For doxorubicin-based experiments, cells were treated with 3 μg/ml of the drug, preliminary defined as a sub-toxic concentration, 24 h after the transfection with sip53 or siCTR and cultured for additional 48 h. Non-transfected (NT) cells were run in parallel.

### Analysis of miR-380-5p and gene expression levels

Total RNA was isolated using Qiagen RNeasy Mini kit (Qiagen, Hilden, Germany) and digested with 20 U RNase-free DNase. miR-380-5p expression levels were analysed using TaqMan® microRNA Assay (P/N 4427975, Applied Biosystems, Foster City, CA, USA), according to the manufacturer’s instruction. Gene expression analysis was carried on randomly primed total RNA using the High Capacity cDNA Reverse Transcription kit (Applied Biosystems). Each gene was amplified using the following TaqMan® Gene expression assays (Applied Biosystems): ATRX (Hs00230877); TEP1 (Hs00200091); and TSPYL5 (Hs00603217). Amplifications were run on the 7900HT Fast Real-Time PCR System (Applied Biosystems). Data were analysed by SDS 2.2.2 software (Applied Biosystems) and, if not otherwise specified, reported as relative quantity (RQ or Log_10_(RQ)) with respect to a calibrator sample using the 2^−ΔΔCt^ method, where Ct represents the threshold cycle. RNU48 snRNA (P/N 4427975) and RNaseP (P/N 4316844) were used as normalisers during the analysis of miR-380-5p and mRNAs expression levels, respectively.

### Western immunoblotting

Forty micrograms of total protein extracts prepared according to standard methods was fractioned by SDS-PAGE, transferred onto nitrocellulose filters and incubated o.n. with primary antibodies: mouse monoclonal antibodies raised against β–actin, PAX8, TNKS, WRAP53; rabbit polyclonal antibodies raised against H2AX, γ- H2AX (S139), p21^Waf1^, POT1, PRKCA (Y124), TEP1 and a goat polyclonal antibody raised against TNKS2 (Abcam, Cambridge, UK); rabbit polyclonal antibodies raised against TERF2, TSPYL5 and the mouse monoclonal raised against p53 (Santa Cruz Biotechnology); rabbit polyclonal antibodies raised against PARP1 and CPP32 (Cell Signaling Technology, Danvers, MA, USA). The filters were then probed with secondary peroxidase-linked whole antibodies (GE Healthcare, Milano, Italy) and detected by Novex® ECL HRP Chemiluminescent detection system (Thermo Fisher). Filters were then subjected to autoradiography. β-Actin (ACTB) was used on each blot to ensure equal protein loading.

### Luciferase assay

To test the effect of miR-380-5p on its predicted target, the pEZX-MT05 vector (GeneCopoeia Inc., Rockville, MD, USA), hosting the whole 3′-UTR of TSPYL5 (GeneBank accession NM_033512.2) downstream the reporter *Gaussia* luciferase (GLuc) gene and containing a second reporter gene (Secreted Alkaline Phosphatase, SEAP) for transfection-normalization, was used. A mutated reporter construct (del43-54) characterized by the deletion of the seed region within the predicted miR-380-5p binding site was generated using the QuikChange II site-directed mutagenesis kit (Agilent Technologies Italia S.p.A., Cernusco S/N, Italy), according to the manufacturer’s instructions, in the presence of the following primer pair: F_5′-ctagctgcatcacttatcagtggtttcatctgtatctctc-3′ and R_5′-gagagatacagatgaaaccactgataagtgatgcagctag-3′. To assess the luciferase activity, STO cells were transfected with gene reporter constructs, in the presence or absence of pre-miR-380-5p or preNeg using Lipofectamine3000^TM^ (Thermo Fisher), according to the manufacturer’s protocol and incubated at 37 °C. GLuc and SEAP enzymatic activities were then measured in the culture media collected at 24, 48 and 72 h after transfection using the Secrete-Pair^TM^ Dual Luminescence Assay Kit (GeneCopoeia Inc.), according to manufacturer’s instructions. For the co-transfection experiments, 24 h after the transfection with pEZX-MT05 and pre-miR-380-5p, cells were transfected with miRNA inhibitors (LNA380 or LNA-Neg) and assessed for luciferase activity 48 h later. PEZX-MT05-expressing cells transfected with single oligomers (preNeg, pre-miR-380-5p, LNA-Neg or LNA380) were run in parallel.

### Target enrichment analysis

Cells (2 × 10^7^) were suspended in RIP buffer (10 mM Tris, pH 7.5; 10 mM KCl; 2 mM MgCl_2_; 1 mM DTT, 100 U/ml RNase inhibitor and a protease inhibitor mixture) for 15 min, homogenized with a Dounce homogenizer and centrifuged at 13,000 rpm at 4 °C for 10 min. Five percent of each solution was set apart to be used as control (Input) for the RT-PCR amplification. The remaining was incubated with IgGA/IgG-coated beads for 1 h at r.t. and centrifuged at 13,000 rpm for 1 min. The supernatants were then mixed with 5 μg of a rat monoclonal anti-human Ago2 antibody (Sigma-Aldrich) and a rabbit polyclonal human IgG (Abcam). After an overnight incubation at 4 °C, samples were re-incubated with IgGA/IgG-coated beads for 1 h at 4 °C, centrifugated at 2500 rpm for 1 min and washed twice with RIP buffer containing 150 mM NaCl and 0.5% Nonidet P-40 and once with buffer containing 400 mM NaCl and 0.5% Nonidet P-40. Finally, the RNA was extracted from the AGO2- and IgG-IP complexes using TRIzol (Thermo Fisher), according to the manufacturer’s instruction. The enrichment for miRNA binding sites was evaluated by RT-PCR using the High Capacity cDNA Reverse Transcription kit (Applied Biosystems). Site #1 was amplified by one-step PCR at 95 °C for 5 min followed by 33 cycles [95 °C 45 s; 57 °C 30 s; 72 °C 30 s] and 72 °C for 5 min using the following forward (5′-cagtccccaaagaggaacaa-3′) and reverse (5′-tcatggtccggggtttatta-3′) primer pair. The fragment corresponding to site #2 was first amplified at 95 °C for 5 min followed by 20 cycles [95 °C 45 s; 57 °C 30 s; 72 °C 30 s] using the following forward (5′-cgtaccttggccttcaatgt-3′) and reverse (5′-cagagaccctgaccacacct-3′) primer pair. One tenth of the amplification reaction was then re-amplified for 22 additional cycles under the same conditions followed by an elongation step at 72 °C for 5 min. The reaction products were resolved by agarose gel electrophoresis. Images were captured by UVI_DOC_ HD5 (UVITec Ltd., Cambridge, UK) and subjected to densitometric analysis using ImageJ 1.46r. The fold-enrichment was calculated after normalization towards Input RNA as the ratio between the mRNA levels detected in AGO2-IP or IgG-IP samples and the mRNA levels in IgG-IP sample from non transfected cells.

### Assessment of telomerase activity, telomere length and C-circle DNA levels

Telomerase activity was measured on 1 μg of protein by the Telomeric Repeat Amplification protocol (TRAP) using the (TRAPeze® kit; Merck S.p.A., Vimodrone, Italy) and quantified according to the manufacturer’s instructions [6]. Telomere length analysis was carried out on Rsa I/Hinf I-digested total DNA upon pulsed-field gel electrophoresis and southern blot, as previously described [6]. DNA-Hind III digest (Bio-Rad Laboratories) was used for size determination of telomere restriction fragments (TRF). Mean telomere length (mTL) was calculated by densitometric analysis (ImageJ 1.46r) as reported by Kimura et al. [11]. C-circle DNA levels were assessed by the CC assay (CCA) [12]. Briefly, genomic DNA was incubated at 30 °C for 8 h in the presence or absence of 7.5 U of φ29 DNA polymerase (New England BioLabs), heat-inactivated at 65 °C for 20 min and dot-blotted. After UV-cross-linking, filters were hybridized with a ^32^P-(CCCTAA)_3_ probe using PerfectHyb Plus hybridization buffer (Sigma-Aldrich), washed, exposed to a PhosphoImager screen and scanned on a Typhoon 8600 Variable Mode Imager (GE Healthcare). Signal intensity was quantified using the VisionWorks LS software (UVP Ltd.). Genomic DNA from ALT-positive U-2 Os and telomerase-positive HeLa cells was included as positive and negative control, respectively.

### Statistical analysis

If not otherwise specified, data have been reported as mean values ± s.d. from at least three independent experiments. Two-sided Student’s *t* test was used to analyse differences between samples. *P* values <0.05 were considered statistically significant.

## Results

### miR-380-5p impairs the growth of DMPM cells and inhibits TA by downregulating TEP1

To unveil possible regulators of TA in human cancer, we focused on miR-380-5p, which was found to be significantly under-expressed in TA-positive compared to TA-negative DMPM tissue specimens or to normal peritoneal specimens [5, 13] by miRNA expression profiling (GSE99362) and subsequent validation by real-time RT-PCR (Additional file 1: Supplementary methods and Figure S1).

Functional studies were carried out in TA-positive DMPM cells (STO, MP8 and MP4), which show negligible endogenous levels of the miRNA compared to human normal mesothelial (MES-F) and to TA-negative, ALT-positive cancer cells (Fig. 1b).

The ectopic expression of mature miR-380-5p (Fig. 1c; Additional file 2: Figure S2A) induced a mild, though significant, reduction of DMPM cell growth over time (Fig. 1d; Additional file 2: Figure S2B), whereas it did not impair the growth of either normal mesothelial or telomerase-negative cancer cells (Additional file 2: Figure S2C). miR-380-5p-mediated DMPM cell growth impairment was associated to a remarkable inhibition of TA (61 ± 1.7%, *P* < 0.01; Fig. 1e) as well as to the accumulation of p53 and p21^waf1^ and the occurrence of apoptosis, as revealed by the accumulation of PARP1 and Caspase-3 cleavage products and of γ-H2AX (Fig. 1f). Moreover, miR-380-5p-dependent inhibition of TA was paralleled by a marked decrease in the expression levels of TEP1, which is responsible for the proper functioning of the holoenzyme (Fig. 1a) [14]—both at mRNA and protein level, in all three TA-positive DMPM cell lines but not in the TA-negative U-2 Os cells (Fig. 1g, h).

**Fig. 2:**
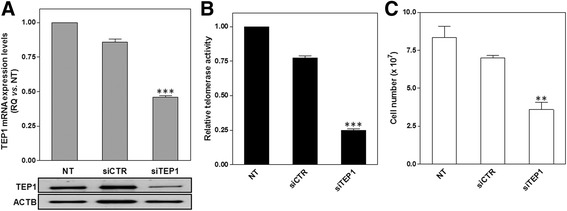
RNAi-mediated depletion of TEP1 recapitulates the biological effects of miR-380-5p. **a** TEP1 mRNA expression levels (upper panel) and protein amounts (lower panel) in STO cells assessed 72 h after the transfection with the indicated siRNAs. Data have been reported as RQ (mean values ± s.d.) in siCTR and siTEP1-transfected vs. NT cells; ***P < 0.001 vs. siCTR-transfected cells. **b** Relative TA in siCTR and siTEP1-transfected STO vs. NT cells (mean values ± s.d.); ***P < 0.001 vs. siCTR-transfected cells. **c** STO cell growth assessed 72 h after siRNA-mediated depletion of TEP1 (number of growing NT, siCTR- and siTEP1-transfected cells; mean values ± s.d.); **P < 0.01 *vs.* siCTR-transfected cells

To test whether the specific downregulation of TEP1 could phenocopy the effects of miR-380-5p, RNAi-mediated depletion of TEP1 was performed in STO cells. Specifically, cells transfected with a siRNA targeting TEP1 (siTEP1) were characterized by a remarkable decrease in TEP1 mRNA and protein amounts (Fig. 2a), which was paralleled by a pronounced inhibition of TA (Fig. 2b) and a significant impairment of cell growth (Fig. 2c), similarly to what was observed at the same time point upon the reconstitution of miR-380-5p. The evidence that the selective depletion of TEP1 reproduced the biological effects observed in miR-380-5p-transfected cells hinted at the possibility that the protein could be a target of the miRNA.

### miR-380-5p directly interacts with the open reading frame of TEP1 and the 3′-UTR of TSPYL5

By an in silico target prediction analysis using miRWalk 2.0 [15], we found a set of genes within the list of putative miR-380-5p targets (Additional file 3: Figure S3A) known for being involved in the maintenance of telomere structure/function (e.g. *POT1*, *TERF2*, *TNKS*, *TNKS2*) as well as in the regulation of the catalytic activity (*TERT*, *WRAP53, PRKCA*) or the expression levels (*TP53*, *PAX8)* of telomerase. However, no miR-380-5p binding sites were predicted within the 3′-untranslated region (UTR) of TEP1 mRNA (Additional file 3: Figure S3A). Moreover, western immunoblotting analyses revealed that, except for a mild reduction in the amounts of PAX8—a transcription factor that transactivates the promoter of both *TERT* and *TERC*—none of the predicted telomere/telomerase-associated targets was found to be downregulated upon the ectopic expression of miR-380-5p (Additional file 3: Figure S3B).

**Fig. 3:**
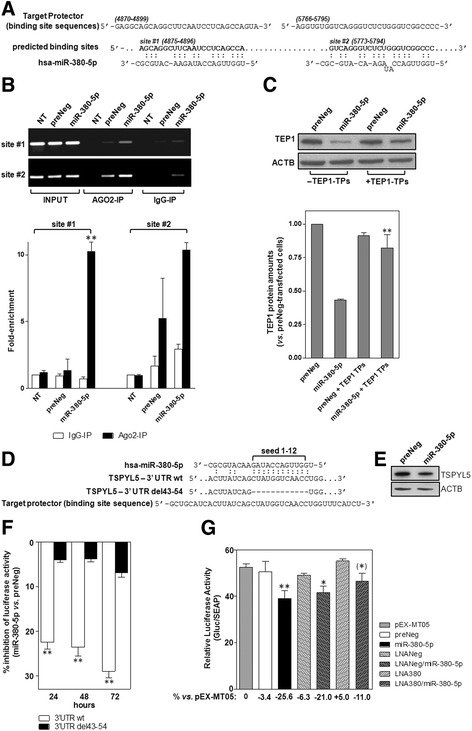
miR-380-5p directly targets the ORF of TEP1 and the 3′-UTR of TSPYL5. **a** Schematic representation of the interaction between miR-380-5p and its predicted TEP1 binding sites. TEP1 sequences used to design target protectors are also shown. *Numbers* in brackets indicate the nucleotide position within TEP1 mRNA (NM_007110.4). **b** Representative RT-PCR showing miR-380-5p target site enrichment. Input: total RNA in cell lysates, AGO2- and IgG-IP: RNA isolated from AGO2- and IgG-immunoprecipitated samples. The graph on the bottom shows TEP1 mRNA enrichment quantification reported as fold-enrichment (mean values ± s.d.) in the indicated immunoprecipitated samples with respect to IgG-IP from NT cells; **P < 0.01. **c** Representative immunobloting showing TEP1 amounts in preNeg- and miR-380-5p-transfected cells ± target protectors. Cropped images of selected proteins are shown. The graph on the bottom shows the quantification of TEP1 protein (relative amounts with respect to preNeg-transfected cells; mean values ± s.d.); **P < 0.01 *vs.* miR-380-5p-transfected cells. **d** Schematic representation of the interaction between miR-380-5p and its predicted binding site within TSPYL5 3′-UTR (NM_033512.2). The mutated sequence (del43-54) bearing a deletion corresponding to miR-380-5p seed and the sequence within TSPYL5 3′UTR used to design the target protector are also shown. (**e**) Representative immunoblotting showing TSPYL5 amounts in miR-380-5p expressing STO cells. Cropped images of selected proteins are shown. **f** Time-course assessment of luciferase activity in cells transfected with the wild-type or mutated (del43-54) reporter constructs in the presence of miR-380-5p (percentage of luciferase activity inhibition in miR-380-5p vs. preNeg-transfected cells; mean values ± s.d.); **P < 0.01 TSPYL5 3’UTRwt vs. TSPYL5 3’UTR del43-54. **g** Assessment of luciferase activity as a function of the different combinations of transfected oligomers. Data have been reported as Gluc/SEAP activity ratio (mean values ± s.d.); *P < 0.05; **P < 0.01 vs. pEX-MT05-transfected cells. *Numbers* under the X-axis indicate the percent reduction (−) or increase (+) of luciferase activity vs. pEX-MT05-expressing cells. *Asterisk* in bracket indicates the level of statistical significance (*P* < 0.05) between LNA380/miR-380-5p and LNANeg/miR-380-5p samples

Nonetheless, two out of five prediction tools within miRWalk database returned the presence of putative miRNA binding sites located within the open reading frame (ORF) of TEP1 and two of them (i.e. site #1 and #2 at positions 4875-4896 and 5773-5794, respectively, GeneBank accession number NM_007110.4, Fig. 3a) were specifically identified by RNA22 v1.0 [16]. To check whether the two sites were recognized by miR-380-5p, their interaction with Argonaute 2 (AGO2), which plays a central role in miRNA-mediated RNA silencing process [17], was examined. Such an analysis revealed a marked enrichment for both target sequences in miR-380-5p- with respect to preNeg-transfected cells (Fig. 3b), with a markedly higher fold-increase for target site #1 *vs.* #2. Target protection experiments were carried out to further verify whether miR-380-5p-mediated downregulation of TEP1 was consequent to the direct miRNA-mRNA interaction at the predicted binding sites. In particular, miR-380-5p precursor was co-transfected with a mix of target protectors (TEP1 TPs), namely single-stranded RNAs designed to specifically bind the miRNA binding sites within TEP1 ORF, thus protecting them from being targeted by miR-380-5p (Fig. 3a). Results revealed a partial, though significant, rescue of TEP1 protein amounts in miR-380-5p-expressing cells in the presence with respect to the absence of TEP1 TPs (Fig. 3c). These data revealed that protection of TEP1 ORF is sufficient to counteract, at least in part, the inhibitory effect of miR-380-5p and, in keeping with target enrichment analyses, indicate that TEP1 is indeed a direct target of the miRNA.

Furthermore, a prominent increase in p53 protein levels was observed upon miR-380-5p reconstitution (Fig. 1f), despite the protein has been reported to be a direct target of the miRNA [18]. This evidence, led us hypothesizing that other than directly targeting the ORF of TEP1, miR-380-5p could also affect the protein function by inhibiting the expression of one or more intermediary factors, the downregulation of which may favour the accumulation of p53, which in turn could impinge on TEP1 [14].

To address this issue, we paid specific attention to testis-specific protein, Y-encoded-like 5 (TSPYL5) gene, a member of TSPY-L family of genes [19]. The gene was predicted by 5/5 algorithms (Additional file 3: Figure S3A) and, according to miRWalk 2.0, bears a highly probable (*P* < 0.0002) miR-380-5p binding site with a 12-nucleotide long seed within the 3′-UTR (Fig. 3d). In keeping with this observation, a reduction of TSPYL5 protein amounts was appreciable in miR-380-5p expressing cells (Fig. 3e). Consequently, reporter gene assays were carried out to check if the gene could be a direct target of the miRNA. Specifically, a significant and time-dependent inhibition (*P* < 0.01) of reporter gene activity was observed upon the ectopic expression of miR-380-5p in cells transfected with the reporter construct bearing the wild-type compared to the deleted form of TSPYL5 3′UTR (Fig. 3d, f). Though apparently modest, the extent of miR-380-5p-mediated inhibition of luciferase activity is consistent with the presence of a single predicted miRNA binding site within the 3′-UTR of the target gene [20] and suggests that TSPYL5 is amenable of targeting by the miRNA. This evidence gained further support also by data showing that miRNA-mediated inhibition of reporter gene activity was significantly counteracted by the co-transfection of miR-380-5p with LNA-380, an inhibitor of the mature form of the miRNA (Fig. 3g).

### The TSPYL5-p53 axis may act as a liaison between miR-380-5p and TEP1

**Fig. 4:**
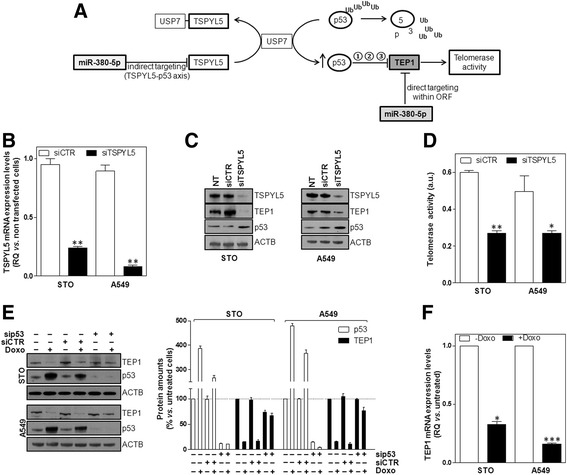
The TSPYL5-p53 axis may act as a liaison between miR-380-5p and TEP1. **a** Schematic representation of the proposed molecular circuitry by which miR-380-5p may interfere with TEP1 expression/function in cancer cells. Other than directly acting on the ORF of TEP1, miR-380-5p may interfere with the gene expression/function via a TSPYL5-p53 axis. It is possible to hypothesize that p53 may impinge on TEP1 levels by (1) transcriptional repression, (2) increased degradation and/or (3) physical interaction (sequestration). *Ub* ubiquitin. **b** Assessment of TSPYL5 expression levels in STO and A549 cells transfected with siCTR or siTSPYL5 (RQ in transfected vs. NT cells; mean values ± s.d.); **P < 0.01 vs*.* siCTR-transfected cells. **c** Representative immunoblottings showing TSPYL5, TEP1 and p53 protein amounts in NT, siCTR- and siTSPYL5-transfected cells. Cropped images of selected proteins are shown. **d** Quantification of TA in siCTR- and siTSPYL5-transfected cells (arbitrary units (au.); mean values ± s.d.); *P < 0.05;**P < 0.01 vs. siCTR-transfected cells. **e** Representative immunoblottings showing TEP1 and p53 protein amounts in STO and A549 cells as a function of the indicated treatment modalities. Cropped images of selected proteins are shown. The graph on the right shows the quantification of protein amounts reported as percentage (mean values ± s.d.) with respect to untreated cells (i.e. no siRNAs and no doxorubicin). **f** Assessment of TEP1 mRNA expression levels in STO and A549 cells in the absence or presence of doxorubicin (RQ in doxorubicin-treated vs*.* untreated cells; mean values ± s.d.); *P < 0.05 ***P < 0.001

It has been recently documented that, through its physical interaction with the ubiquitin specific protease 7, TSPYL5 leads to p53 protein level reduction and, consequently, to the impairment of p53-target gene regulation [19]. As such an event may have a role in regulating TA [14, 21], we envisaged a molecular circuitry by which miR-380-5p-mediated downregulation of TSPYL5 triggers p53 accumulation, which in turn may impinge on TEP1 function and may result in TA inhibition (Fig. 4a). The existence of such miR-380-5p-TSPYL5-TEP1 axis was confirmed in the TA-positive A549 cells, where the functional interplay between TSPYL5 and p53 was firstly documented [19].

Specifically, A549 cells are characterized by barely detectable levels of endogenous miR-380-5p (Fig. 1b) and, similarly to what observed in DMPM cells, the expression of miR-380-5p induced a significant and time-dependent slow down of cell growth (Additional file 4: Figure S4 A-B), which was accompanied by a remarkable reduction of TEP1 and TSPYL5 mRNA and proteins as well as by the accumulation of p53 (Additional file 4: Figure S4C-D). Of note, miR-380-5p failed to affect TSPYL5 RNA and protein levels and, consequently, of p53 in the TA-negative U-2 Os cell line (Additional file 4: Figure S4E-F), which showed barely detectable levels of endogenous TSPYL5 protein (Additional file 4: Figure S4E-F) compared to TA-positive DMPM and A549 cells.

The involvement of TSPYL5 in mediating miR-380-5p biological effects was hence explored by phenocopy experiments. Specifically, a remarkable reduction of TSPYL5 mRNA and protein amounts was observed in both STO and A549 cells after the transfection with a siRNA targeting TSPYL5 (siTSPYL5) (Fig. 4b, c). In addition, the selective depletion of TSPYL5 led to p53 accumulation and, similarly to what observed upon miR-380-5p reconstitution, to TEP1 protein reduction and TA inhibition (Fig. 4c, d). This evidence indicates that the depletion of TSPYL5 and the consequent accumulation of p53 may contribute, at least in part, to the direct effect of the miRNA on TEP1. In fact, we found that the inhibitory effect on TEP1 expression levels exerted by the miRNA was partly counteracted by the selective depletion of p53 (Additional file 4: Figure S4C and G). In addition, TSPYL5 target protection counteracted the inhibitory effect of miR-380-5p on the target and resulted in a reduced accumulation of p53 and in a rescue, though partial, of TEP1 protein amounts (Additional file 4: Figure S4 H and I).

The causative role of p53 in the regulation of TEP1 levels gained further support by the evidence that the remarkable accumulation of p53 caused by the exposure of STO and A549 cells to doxorubicin was accompanied by a dramatic reduction of TEP1 protein amounts (Fig. 4e), which instead were not remarkably affected upon silencing of p53 (Fig. 4e). These observations indicate that p53 may be actually involved in the control of TEP1 expression, as also suggested by the finding that doxorubicin induced a significant down-modulation of TEP1 mRNA levels in p53-proficient STO and A549 cells (Fig. 4f).

### Long-term miR-380-5p transfectants exhibit features reminiscent of ALT

**Fig. 5:**
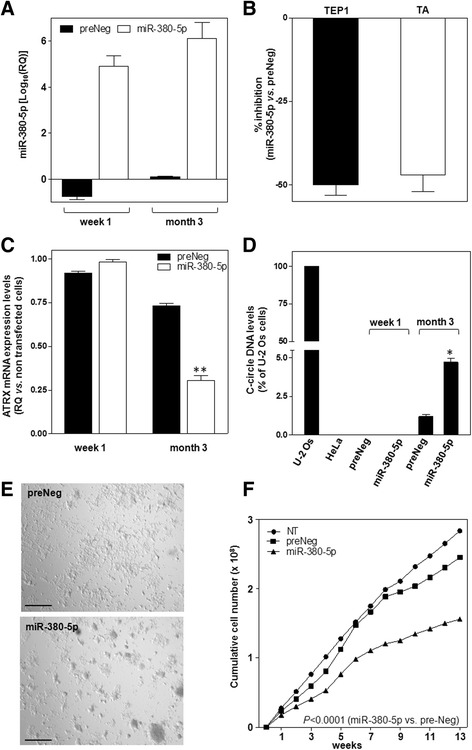
miR-380-5p mixes up the phenotype of DMPM cells. **a** Assessment of miR-380-5p expression levels upon weakly transfection of STO cells with preNeg or miR-380-5p precursor (Log_10_(RQ) with respect to NT cells; mean values ± s.d.). **b** Quantification of TEP1 mRNA levels and of TA in miR-380-5p expressing cells at month 3. Data have been reported as percentage inhibition (mean values ± s.d.) in miR-380-5p- vs. preNeg-transfected cells. **c** Assessment of ATRX expression levels (RQ in preNeg- and miR-380-5p-transfected vs. NT cells; mean values ± s.d.); **P < 0.01 for miR-380-5p vs. preNeg. **d** Quantification of C-circle DNA levels in preNeg- and miR-380-5p-transfected cells at the indicated time points (percentage of c-circle DNA levels in each sample with respect to U-2 Os cells; mean values ± s.d.); HeLa: negative control; *P < 0.05 for miR-380-5p vs. preNeg at month 3. **e** Representative photomicrographs showing the morphology of preNeg- and miR-380-5p-transfected STO cells. Original magnification: ×4; Scale bar: 100 μm. **f** Growth curves of NT, preNeg- and miR-380-5p-transfected STO cells. Data have been reported as cumulative number of growing cells over a 3-month period. The level of statistical significance for the comparison between preNeg and miR-380-5p groups has been assessed by two-way ANOVA

In accordance with the paradigm that a lag period is required to induce sufficient telomere shortening to impair cancer cell growth in the presence of inhibited TA [7], long-term reconstitution of miR-380-5p was pursued. Similarly to what observed in the short-term setting, after 3 months of weakly reiterated transfections with miR-380-5p, STO cells showed high levels of mature miR-380-5p (Fig. 5a) as well as a remarkable decrease in TEP1 mRNA expression levels and TA inhibition (Fig. 5b). Nevertheless, no evident telomere exhaustion was appreciable over this period of time (Table 1). Contrarily, upon 3 months of persistent reconstitution of miR-380-5p, a slight but significant increase in mean telomere length (about +1 Kb) was observed (Table 1). In addition, long-term miR-380-5p transfectants showed a significant reduction in the expression levels of ATRX (Fig. 5c) as well as detectable levels of C-circle DNA (Fig. 5d), two molecular markers associated with ALT activity [2, 22]. In addition, long-term STO cells expressing miR-380-5p were characterized by altered growing properties, in that compared to preNeg-transfected cells, they prevalently formed clusters with a remarkable component of vertical growth (Fig. 5e). However, despite these alterations in the growing properties, no growth arrest or massive culture loss was observed over the 3-month period of miR-380-5p reconstitution. Instead, miR-380-5p-expressing cells continued to grow in culture, though at significantly reduced rate compared to control cells (Fig. 5f).

**Table 1:** Quantification of mean telomere length (mTL) in STO cells transfected with preNeg or miR-380-5p

	mTL (Kb) week 1	mTL (Kb) month 3	*P* ^a^
preNeg	3.35 ± 0.17	3.50 ± 0.20	n.s.
miR-380-5p	3.29 ± 0.15	4.28 ± 0.30	<0.005
*P* ^b^	n.s.	*P* < 0.01	

*n.s* not significant

^a^month 3 *vs.* week 1

^b^miR-380-5p *vs.* preNeg (Student’s t-test)

## Discussion

In the present study, we reported that the ectopic reconstitution of miR-380-5p in DMPM cells with active telomerase and negligible levels of endogenous miRNA results in TA inhibition and in cell growth impairment. We documented for the first time that such an effect was the result of a molecular circuitry converging on TEP1, where the miRNA was able to target it directly within the ORF and indirectly via TSPYL5 down-modulation and p53 accumulation.

The evidence that miR-380-5p may reasonably play a role in regulating TA via a TSPYL5-p53 axis, other than by directly targeting TEP1, gains support by early evidence showing that p53 may repress telomerase by interacting with TEP1 and preventing it from correctly assembly with the holoenzyme [14]. In addition, the over-expression of wild-type p53 has been shown to inhibit TA in several cancer cell lines by causing TERT transcriptional repression through an indirect mechanism involving p21^waf1^ [21], which indeed accumulated in cells ectopically expressing miR-380-5p (Fig. 1f). In keeping with our findings is the evidence that telomerase reactivation during cancer cell immortalization may occur through the impairment of the p53-p21^waf1^ pathway caused by the overexpression of miR-296, which was found up-regulated in telomerase-positive cancer cells [23] as well as that miR-375-mediated suppression of multiple oncogenic factors resulted in TA inhibition as a consequence of p53-p21^waf1^ accumulation in HPV-associated cancer cells [24]. These findings are in keeping with recent observations showing that the selective depletion of TSPYL5 in A549 cells resulted in a marked accumulation of p21^waf1^ associated with a reduced activation of the PI3K/AKT cell growth/survival pathway [25] and that the ectopic expression of TSPYL5 prevented the cell growth arrest induced by the acute overexperession of RAS^V12^ in TERT-immortalized human diploid fibroblast selectively depleted for p16^INK4a^ [19].

Our findings also showed that long-term miR-380-5p transfectants, despite characterized by a remarkable downregulation of TEP1 expression levels and TA inhibition, did not undergo telomere attrition. Oppositely, these cells, which continued to grow in cultures though at slower rate compared to parental cells, where characterized by reduced expression of ATRX and by the appearance of C-circle DNA, two molecular features usually associated with ALT activity [2].

This event is anything but trivial. In fact, telomerase is activated in the great majority of human cancers [7], including DMPM [5], and Imetelstat has been the first inhibitor of telomerase catalytic activity to enter clinical trials for different malignancies [26]. In addition, other than being activated in a subset of human tumours with no detectable TA, ALT may co-exist with telomerase due to intratumoural heterogeneity or the spontaneous activation of both TMM within the same tumour cells [1]. Consequently, treatment of these tumours with telomerase inhibitors could favour the clonal expansion of ALT cell subpopulations that will be refractory to anti-telomerase therapies. Moreover, it is plausible to hypothesize that ALT, which may be considered a less readily controllable TMM compared to telomerase [27], may likely emerge in telomerase-positive tumours upon prolonged exposure to enzyme inhibitors, hence representing a threat to the clinical efficacy of this class of therapeutic agents. Even if such a hypothesis has not been confirmed in the clinical setting yet, it gains support by preclinical evidence showing that T-cell lymphomas arising in telomerase knocked-out mice enter a period of slow growth and then emerge with ALT features [28]. Moreover, it has been observed that laryngeal cancer cells depleted for TERT continue to proliferate in culture, though at slower rate compared to parental cells, despite a marked inhibition of TA, and, after 30 population doublings, show a significantly higher mean telomere length associated with evidence of ALT [29]. In addition, the RNAi-mediated co-depletion of histone chaperone anti-silencing factors 1a and 1b in TERT-immortalized lung fibroblasts and in a sub-line of HeLa cells characterized by long telomeres resulted in TA inhibition and the concomitant appearance of ALT-associated hallmarks [30].

## Conclusions

The comprehension of the molecular and cellular features of DMPM is of utmost importance for the fruitful management of the disease, especially in patients who fail standard treatments and have a poor prognosis due to the lack of effective alternative therapeutic options [31, 32]. In this context, TMM have been proposed to represent a reservoir of candidate targets for the development of novel therapeutic interventions for the disease [32].

Limited information is currently available on the functional role of specific miRNAs in DMPM [13], and a handful of miRNAs have been identified thus far for having a role in the regulation of telomere maintenance in cancer [1, 23, 24, 33–36]. Here, we have provided evidence that miR-380-5p may interfere with TA and leads to cell growth impairment and induction of apoptosis in relevant models of DMPM. In addition, we have shown that long-term ectopic reconstitution of miR-380-5p leads to the emergence of cells with features that, at least in part, are reminiscent of an ALT phenotype (that here we refer to as “ALT-like”) that could provide DMPM cells with a compensatory mechanism to ensure continuous cell growth even in a context of partially inhibited TA. This observation deals with a major clinical challenge that telomerase-based therapeutic agents have to face, that is the need of a continuous, long lasting treatment to achieve clinically relevant therapeutic responses [7]. This may in turn results in toxicities which would require off treatment periods (hence loss of achieved benefits) and/or in the emergence of subpopulations of “ALT-reverting” cells that do not respond anymore to telomerase inhibitors.

A genome-wide analysis of the molecular alterations occurring in DMPM cells upon the long-term restoration of miR-380-5p could be instrumental for the identification of specific factors responsible for the emergence of the “ALT-like” phenotype to be validated as therapeutic targets. This information may hence furnish the biological rationale for the development of novel therapeutic interventions not only to be used in combination with inhibitors of the canonical function of telomerase in TA-positive tumours [37] but also to be tested in ALT-naïve tumour models.

## Additional files

Additional file 1: Figure S1. miR-380-5p is significantly under-expressed in telomerase-positive DMPM tissue specimens. Description of data: (**A**) miR-380-5p endogenous expression levels assessed by miRNA expression profiling. Data have been reported as log_2_-transformed normalized signal intensities for miR-380-5p in each specimen. Horizontal lines represent the mean values of miR-380-5p expression levels in each group (normal vs. TA-positive *vs.* TA-negative specimens). (**B**) Real-time RT-PCR validation of endogenous miR-380-5p expression levels in normal samples, in TA-positive and TA-negative DMPM tissue specimens. Data have been reported as Log_10_(RQ) (mean values ± s.d.) with respect to the average miRNA expression levels detected in normal samples, using the 2^−ΔΔCt^ method. **P < 0.01 *vs.* TA-negative specimens. (PDF 72 kb)

Additional file 2: Figure S2. miR-380-5p impairs the growth of DMPM cells. Description of data: (**A**) Time-course assessment of miR-380-5p expression levels in preNeg- and miR-380-5p-transfected MP8 and MP4 cells (Log_10_(RQ) with respect to NT cells; mean values ± s.d.). (**B**) Growth curves of NT, preNeg- and miR-380-5p-transfected MP8 and MP4 cells (number of growing cells; mean values ± s.d.); *P < 0.05; **P < 0.02 miR-380-5p vs*.* preNeg. (**C**) Assessment of miR-380-5p expression levels (white bars) and cell growth (black bars) after a 96-h transfection of MES-F and U-2 Os cells with preNeg or miR-380-5p precursor. Data have been reported as Log_10_(RQ) for miRNA expression levels (left Y-axis) and as the percentage of growing cells (right Y-axis) with respect to NT cells (mean values ± s.d.). (TIF 1087 kb)

Additional file 3: Figure S3. In silico prediction analysis of putative miR-380-5p target genes by miRWalk 2.0. Description of data: (**A**) By the predicted target module of miRWalk 2.0—a comprehensive database that provides indications on predicted and validated binding sites on miRNA target genes [14]—we obtained a combined information on putative miR-380-5p binding sites within the 3′UTRs of human RefSeq mRNAs in terms of union of the predictions generated by five distinct algorithms (i.e. miRWalk 2.0; miRanda-rel2010; miRMap; RNA22v2 and Targetscan6.2). (**B**) Representative western immunoblotting showing the amounts of protein encoded by predicted miR-380-5p target genes in STO cells transfected with preNeg or miR-380-5p. Target proteins have been selected among those known to play a role in TMM and reported in panel A. Cropped images of selected proteins are shown. (TIF 432 kb)

Additional file 4: Figure S4. Effects of miR-380-5p reconstitution on A549 lung adenocarcinoma cells. Description of data: (**A**) Assessment of miR-380-5p expression levels in preNeg and miR-380-5p-transfected cells (Log_10_(RQ) vs. NT cells; mean values ± s.d.). (**B**) Growth curves of NT, preNeg- and miR-380-5p-transfected cells (number of growing cells; mean values ± s.d.); **P < 0.02. (**C**) Assessment of TEP1 (black bars) and TSPYL5 (white bars) mRNA expression levels in p53 proficient (siCTR) and p53-depleted (sip53) cells ectopically expressing miR-380-5p (Log_10_(RQ) *vs.* NT cells; mean values ± s.d.); *P < 0.05 *vs.* siCTR-transfected cells. (**D**) Representative immunoblotting showing TSPYL5, TEP1 and p53 protein amounts in NT, preNeg- and miR-380-5p-transfected A549 cells. Cropped images of selected proteins are shown. (**E**) Assessment of TSPYL5 mRNA expression levels in preNeg- and miR-380-5p-transfected U-2 Os cells (RQ *vs.* NT cells; mean values ± s.d.). (**F**) Representative immunoblotting showing TSPYL5 and p53 protein amounts in NT, preNeg- and miR-380-5p-transfected U-2 Os cells. Cropped images of selected proteins are shown. (**G**) Representative immunoblotting showing p53, TEP1 and TSPYL5 protein levels in p53 proficient (siCTR) and p53-depleted (sip53) cells ectopically expressing miR-380-5p. Cropped images of selected proteins are shown. The graph on the right shows the quantification of TEP1 (black bars) and TSPYL5 (white bars) protein amounts as a function of the different transfected oligomers (relative quantity *vs.* NT cells; mean values ± s.d.); *P < 0.05 *vs.* siCTR-transfected cells. (**H**) Representative immunobloting showing TSPYL5, p53 and TEP1 amounts in preNeg- and miR-380-5p-transfected cells ± target protector (TSPYL5 TP). Cropped images of selected proteins are shown. (**I**) Quantification of TSPYL5 (white bars), TEP1 (black bars) and p53 (grey bars) protein amounts in preNeg- and miR-380-5p-transfected cells ± TSPYL5 TP (relative amounts with respect to preNeg-transfected cells; mean values ± s.d.); **P < 0.01 *vs.* miR-380-5p-transfected cells. (TIF 1515 kb)

## Abbreviations

AGO2: Argonaute 2
ALT: Alternative lengthening of telomeres
APB: ALT-associated promyelocytic leukaemia bodies
ATRX: ATRX chromatin remodeler
DICER1: Dicer 1 ribonuclease III
DMPM: Diffuse malignant peritoneal mesothelioma
HPV: Human papilloma virus
miRNA: microRNA
mTL: Mean telomere length
ORF: Open reading frame
PAX8: Paired box 8
POT1: Protection of telomere 1
PRKCA: Protein kinase C alpha
RISC: RNA-induced silencing complex
RNAi: RNA interference
siRNA: Small interfering RNA
TA: Telomerase activity
TEP1: Telomerase associated protein 1
TERC: Telomerase RNA component
TERF2: Telomeric repeat binding factor 2
TERT: Telomerase reverse transcriptase
TMM: Telomere maintenance mechanisms
TNKS: Tankyrase 1
TNKS2: Tankyrase 2
TP53: Tumour protein p53
TPs: Target protectors
TRF: Telomere restriction fragment
TSPYL5: Testis-specific Y-encoded-like 5
UTR: Untranslated region
WRAP53: WD repeat containing antisense to TP53
