# Supplementary material for: miR-380-5p-mediated repression of TEP1 and TSPYL5 interferes with telomerase activity and favours the emergence of an “ALT-like” phenotype in diffuse malignant peritoneal mesothelioma cells
Source: J Hematol Oncol. 2017 Jul 17;10:140. doi: 10.1186/s13045-017-0510-3 (PMC5513108; doi:10.1186/s13045-017-0510-3)
Supplement: Supplementary file 1 — miR-380-5p is significantly under-expressed in telomerase-positive DMPM tissue specimens. Description of data: (A) miR-380-5p endogenous expression levels assessed by miRNA expression profiling. Data have been reported as log2-transformed normalized signal intensities for miR-380-5p in each specimen. Horizontal lines represent the mean values of miR-380-5p expression levels in each group (normal vs. TA-positive vs. TA-negative specimens). (B) Real-time RT-PCR validation of endogenous miR-380-5p expression levels in normal samples, in TA-positive and TA-negative DMPM tissue specimens. Data have been reported as Log10(RQ) (mean values ± s.d.) with respect to the average miRNA expression levels detected in normal samples, using the 2−ΔΔCt method. **P < 0.01 vs. TA-negative specimens. (PDF 72 kb) [file 13045_2017_510_MOESM1_ESM.pdf]

Additional File 1: Supplementary methods and Figure S1

MiRNA expression profiling

MiRNA expression profiling was carried out on a series of 50 DMPM tissue specimens obtained from patients treated at our Institute between 1997 and 2009, 1 cell line derived from the tumour of a DMPM patient and in 6 normal samples (5 normal peritoneum specimens from patients who underwent surgery for non-oncologic disease and 1 human normal mesothelial cell line, MES-F). The study was approved by the Institutional Review Board and each patient provided written informed consent to donate to INT the leftover tissue after diagnostic and clinical procedures [5,13]. DMPM specimens were defined as TA-positive (n=25) or TA–negative (n=25) according to a previous screening for telomerase activity [5]. MiRNA expression profiling was carried out on the Illumina Human v2 microRNA expression beadchip (GPL8179) and raw data analyzed using Illumina BeadStudio v3.1.3 (Gene Expression Module v3.3.8). Methodological details may be found at GEO [GSE99362, https://www.ncbi.nlm.nih.gov/geo/query/acc.cgi?acc=GSE99362].

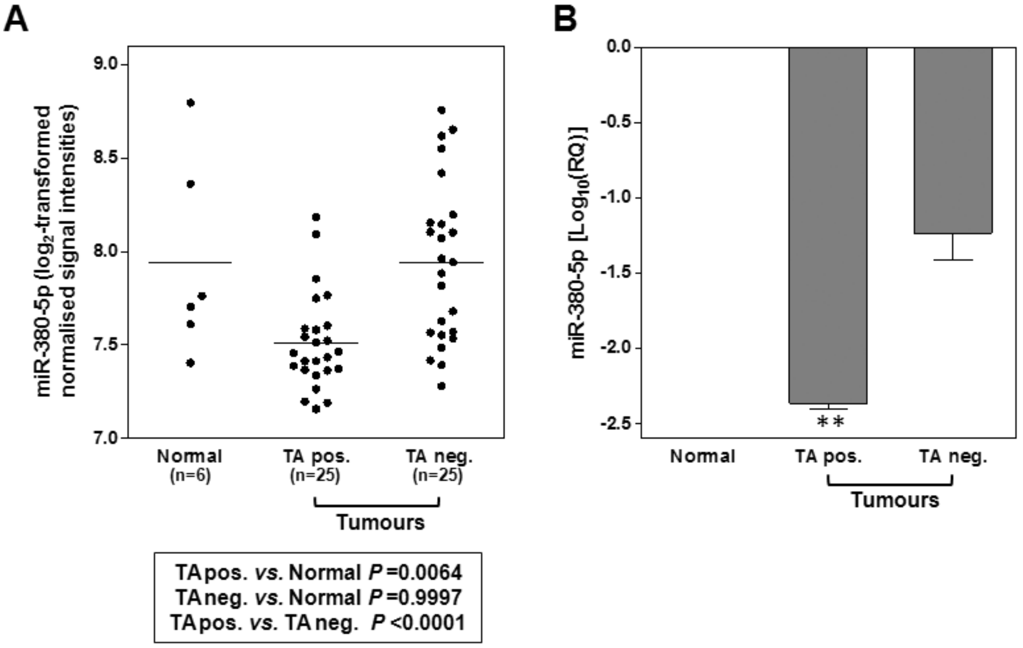

Figure S1. miR-380-5p is significantly under-expressed in telomerase-positive DMPM tissue specimens. **(A)** miR-380-5p endogenous expression levels assessed by miRNA expression profiling. Data have been reported as log_2-transformed normalized signal intensities for miR-380-5p in each specimen. Horizontal lines represent the mean values of miR-380-5p expression levels in each group (normal *vs.* TA-positive *vs.* TA-negative specimens). **(B)** Real-time RT-PCR validation of endogenous miR-380-5p expression levels in normal samples, in TA-positive and TA-negative DMPM tissue specimens. Data have been reported as Log_{10}(RQ) (mean values ± s.d.) with respect to the average miRNA expression levels detected in normal samples, using the $2^{-\Delta\Delta Ct}$ method. **P< 0.01 *vs.* TA-negative specimens (Student's t-test).
