# Supplementary figures and images for: miR-380-5p-mediated repression of TEP1 and TSPYL5 interferes with telomerase activity and favours the emergence of an “ALT-like” phenotype in diffuse malignant peritoneal mesothelioma cells
Source: J Hematol Oncol. 2017 Jul 17;10:140. doi: 10.1186/s13045-017-0510-3 (PMC5513108; doi:10.1186/s13045-017-0510-3)

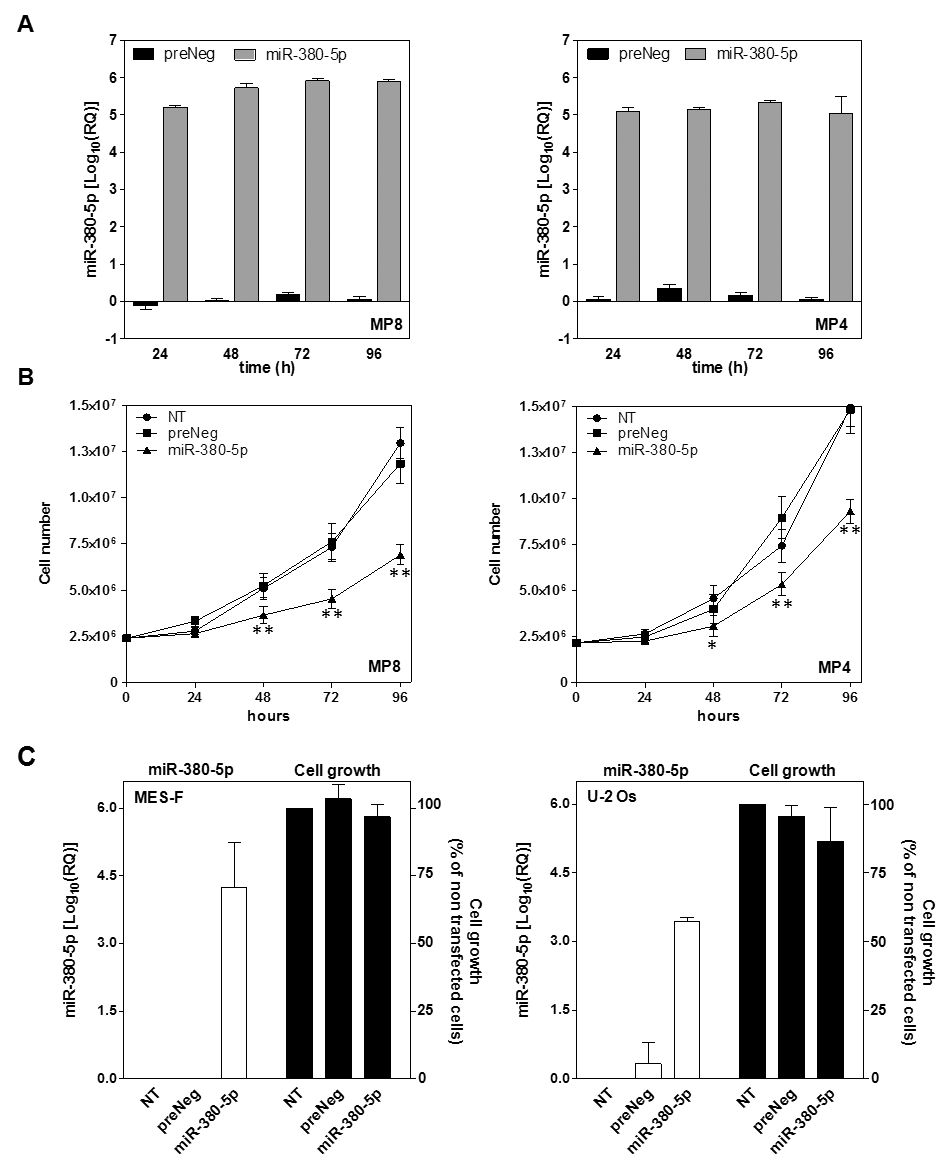

Supplement: Supplementary file 2 — miR-380-5p impairs the growth of DMPM cells. Description of data: (A) Time-course assessment of miR-380-5p expression levels in preNeg- and miR-380-5p-transfected MP8 and MP4 cells (Log10(RQ) with respect to NT cells; mean values ± s.d.). (B) Growth curves of NT, preNeg- and miR-380-5p-transfected MP8 and MP4 cells (number of growing cells; mean values ± s.d.); *P < 0.05; **P < 0.02 miR-380-5p vs. preNeg. (C) Assessment of miR-380-5p expression levels (white bars) and cell growth (black bars) after a 96-h transfection of MES-F and U-2 Os cells with preNeg or miR-380-5p precursor. Data have been reported as Log10(RQ) for miRNA expression levels (left Y-axis) and as the percentage of growing cells (right Y-axis) with respect to NT cells (mean values ± s.d.). (TIF 1087 kb) [file 13045_2017_510_MOESM2_ESM.tif]

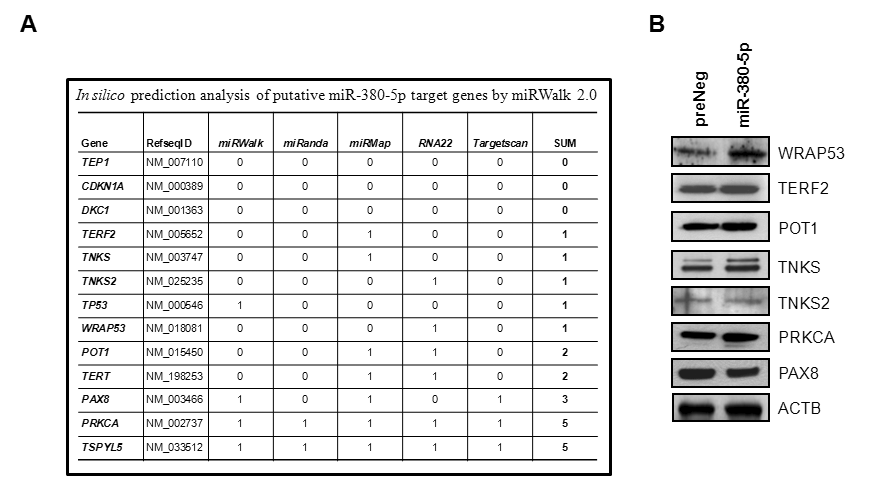

Supplement: Supplementary file 3 — In silico prediction analysis of putative miR-380-5p target genes by miRWalk 2.0. Description of data: (A) By the predicted target module of miRWalk 2.0—a comprehensive database that provides indications on predicted and validated binding sites on miRNA target genes [14]—we obtained a combined information on putative miR-380-5p binding sites within the 3′UTRs of human RefSeq mRNAs in terms of union of the predictions generated by five distinct algorithms (i.e. miRWalk 2.0; miRanda-rel2010; miRMap; RNA22v2 and Targetscan6.2). (B) Representative western immunoblotting showing the amounts of protein encoded by predicted miR-380-5p target genes in STO cells transfected with preNeg or miR-380-5p. Target proteins have been selected among those known to play a role in TMM and reported in panel A. Cropped images of selected proteins are shown. (TIF 432 kb) [file 13045_2017_510_MOESM3_ESM.tif]

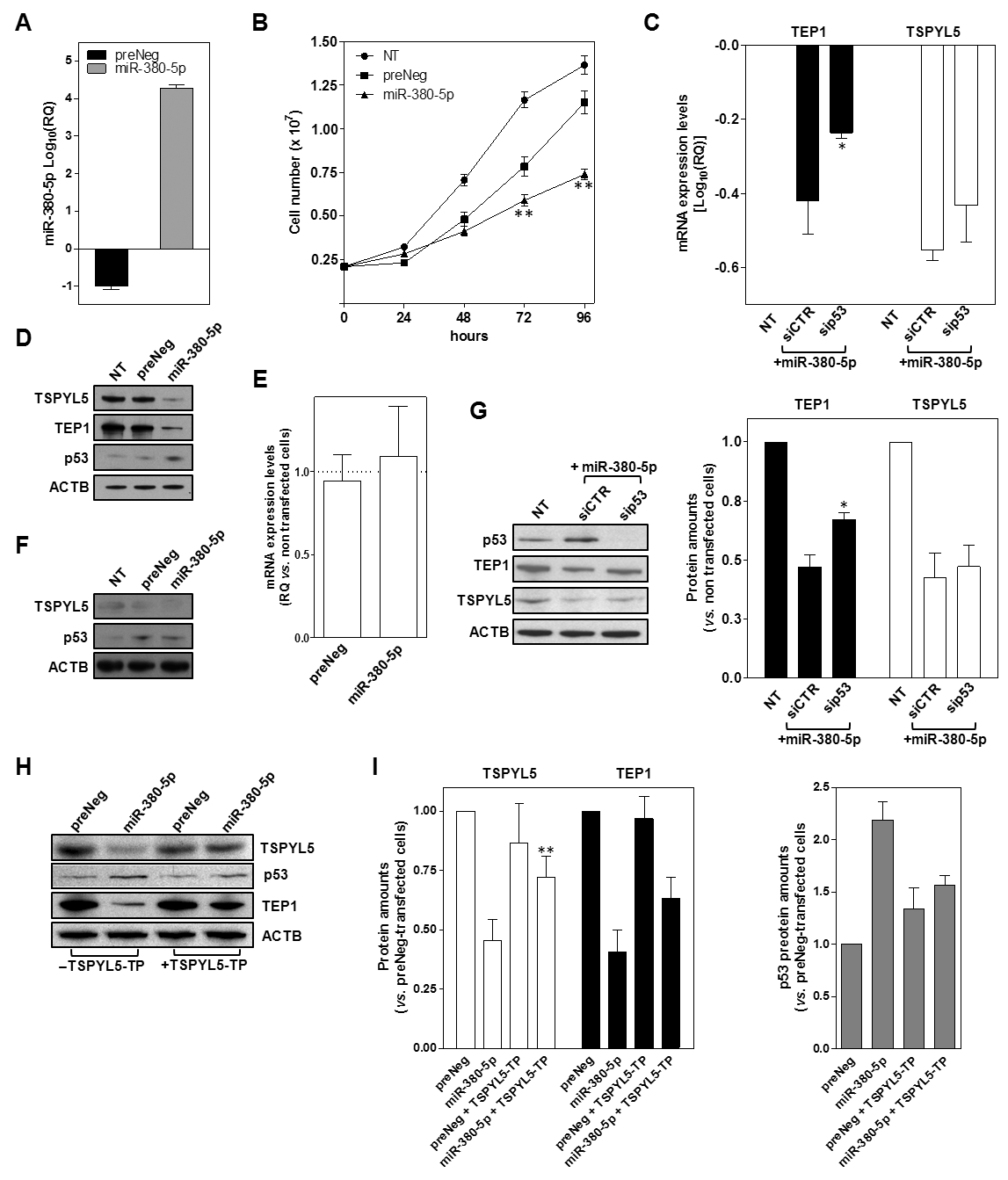

Supplement: Supplementary file 4 — Effects of miR-380-5p reconstitution on A549 lung adenocarcinoma cells. Description of data: (A) Assessment of miR-380-5p expression levels in preNeg and miR-380-5p-transfected cells (Log10(RQ) vs. NT cells; mean values ± s.d.). (B) Growth curves of NT, preNeg- and miR-380-5p-transfected cells (number of growing cells; mean values ± s.d.); **P < 0.02. (C) Assessment of TEP1 (black bars) and TSPYL5 (white bars) mRNA expression levels in p53 proficient (siCTR) and p53-depleted (sip53) cells ectopically expressing miR-380-5p (Log10(RQ) vs. NT cells; mean values ± s.d.); *P < 0.05 vs. siCTR-transfected cells. (D) Representative immunoblotting showing TSPYL5, TEP1 and p53 protein amounts in NT, preNeg- and miR-380-5p-transfected A549 cells. Cropped images of selected proteins are shown. (E) Assessment of TSPYL5 mRNA expression levels in preNeg- and miR-380-5p-transfected U-2 Os cells (RQ vs. NT cells; mean values ± s.d.). (F) Representative immunoblotting showing TSPYL5 and p53 protein amounts in NT, preNeg- and miR-380-5p-transfected U-2 Os cells. Cropped images of selected proteins are shown. (G) Representative immunoblotting showing p53, TEP1 and TSPYL5 protein levels in p53 proficient (siCTR) and p53-depleted (sip53) cells ectopically expressing miR-380-5p. Cropped images of selected proteins are shown. The graph on the right shows the quantification of TEP1 (black bars) and TSPYL5 (white bars) protein amounts as a function of the different transfected oligomers (relative quantity vs. NT cells; mean values ± s.d.); *P < 0.05 vs. siCTR-transfected cells. (H) Representative immunobloting showing TSPYL5, p53 and TEP1 amounts in preNeg- and miR-380-5p-transfected cells ± target protector (TSPYL5 TP). Cropped images of selected proteins are shown. (I) Quantification of TSPYL5 (white bars), TEP1 (black bars) and p53 (grey bars) protein amounts in preNeg- and miR-380-5p-transfected cells ± TSPYL5 TP (relative amounts with respect to preNeg-transfected cells; mean values ± s [file 13045_2017_510_MOESM4_ESM.tif]
